# Preliminary results of the cross-sectional associations of sedentary behavior and physical activity with serum brain-derived neurotrophic factor in adults with coronary heart disease

**DOI:** 10.1038/s41598-022-23706-8

**Published:** 2022-11-16

**Authors:** Antje Ullrich, Kristin Wenzel, Martin Bahls, Lisa Voigt, Stephanie Könemann, Marcus Dörr, Susanne Wurm, Sabina Ulbricht

**Affiliations:** 1 Department of Prevention Research and Social Medicine, Institute for Community Medicine, University Medicine Greifswald, Walther-Rathenau-Str. 48, 17475 Greifswald, Germany; 2 German Centre for Cardiovascular Research (DZHK), Partner Site Greifswald, Greifswald, Germany; 3 Department of Internal Medicine B, University Medicine Greifswald, Greifswald, Germany

**Keywords:** Cardiology, Risk factors

## Abstract

This is the first study to analyze the association of accelerometer-measured patterns of habitual physical activity (PA) and sedentary behavior (SB) with serum BDNF in individuals with coronary heart disease. A total of 30 individuals (M = 69.5 years; 80% men) participated in this pre-post study that aimed to test a multi-behavioral intervention. All participants underwent standardized measurement of anthropometric variables, blood collection, self-administered survey, and accelerometer-based measurement of PA and SB over seven days. Serum BDNF concentrations were measured using enzyme-linked immunosorbent assay kit. We applied separate multiple linear regression analysis to estimate the associations of baseline SB pattern measures, light and moderate-to-vigorous PA with serum BDNF (n = 29). Participants spent 508.7 ± 76.5 min/d in SB, 258.5 ± 71.2 min/d in light PA, and 21.2 ± 15.2 min/d in moderate-to-vigorous PA. Per day, individuals had 15.5 ± 3.2 numbers of 10-to-30 min bouts of SB (average length: 22.2 ± 2.1 min) and 3.4 ± 1.2 numbers of > 30 min bouts of SB (average length: 43.8 ± 2.4 min). Regression analysis revealed no significant associations between any of the accelerometer-based measures and serum BDNF. The findings of this study did not reveal an association of accelerometer-measured PA and SB pattern variables with serum BDNF in individuals with coronary heart disease. In addition, our data revealed a considerable variation of PA and SB which should be considered in future studies.

## Introduction

Being physically inactive, characterized as a combination of low levels of moderate-to-vigorous physical activity (MVPA) and high amounts of sedentary behavior (SB), has been linked to detrimental effects on physical^1,2^ and brain health^3–5^. Regular physical activity (PA) has been shown to increase brain functions such as cognition, memory, and attention^3,4^. Evidence suggests that brain-derived neurotrophic factor (BDNF) may play a fundamental role in mediating this association.^6,7^ Aside from its important role in the development, maintenance, and plasticity of the neurons and the regulation of energy homeostasis, BDNF is also produced peripherally (i.e., skeletal muscle, adipose tissue)^8^. There is increasing evidence that PA stimulates the synthesis of BDNF due to its function as a contraction-inducible protein in the skeletal muscle^7^. Given the amount of studies that examined the effects of PA on BDNF, a distinction between different paradigms is necessary^7,9^. While studies have consistently emphasized that acute PA increases concentrations of BDNF in healthy adults^7,9,10^, exercise training seems to have varying effects on resting BDNF concentrations^7,10^. In healthy adults, the brain seems to contribute to increases in BDNF during exercise, but the source of circulating BDNF remains controversial^8,11^.

From a public health perspective, it is important to consider that individuals spend the majority of the day in SB and comparatively little time in MVPA. The lack of PA characterized by a sedentary lifestyle increases with age^12^ and accelerates the development of chronic diseases such as cardiovascular disease (CVD)^13^. Moreover, individuals with CVD typically report low levels of MVPA and high levels of SB compared to healthy controls^14–16^, which may enhance the risk for CVD progression and mortality^17,18^.

In the past years, research focus has shifted from total time spent sitting to the importance of considering an accumulation pattern of SB^19,20^, but there is insufficient information about distinct patterns of PA and SB, particularly in individuals with CVD. In a community-dwelling sample of 5638 American women from the Objective Physical Activity and Cardiovascular Health (OPACH) study, results showed that women who accumulated sedentary time in longer, uninterrupted sedentary bouts seem to have a higher risk for CVD compared to those who had shorter sedentary bout durations^16^. Further, in a sample of 248 Dutch adults, 131 individuals with CVD showed significantly more prolonged uninterrupted sedentary bouts compared to 117 healthy controls^14^. In particular, prolonged periods of sitting have been found to have adverse effects on vascular function and blood pressure^21^.

Recent evidence has identified BDNF as a protective factor in the pathogenesis of CVD due to its involvement in cardiac processes such as angiogenesis, vascular growth, and survival of cardiomyocytes^22–24^. In line with these findings, high levels of BDNF were found to be associated with reduced risk of CVD and mortality^25^. Furthermore, low BDNF levels seem to be associated with increased risk of future adverse cardiovascular events^26^ and mortality in individuals with angina pectoris^27^ and heart failure^28^.

Therefore, it is important to examine how habitual PA and SB are associated with resting BDNF levels. Studies investigating the effect of different types of accelerometer-measured habitual PA and SB (e.g., total time spent sedentary, or different lengths of sedentary bouts) with resting BDNF are rare and the findings are inconsistent. The controversial results of current research might be due to differences regarding sample characteristics, medical conditions, and age group^29–34^. In an adult population, one study did not find significant associations between habitual PA (light physical activity [LPA], MVPA) and resting BDNF^33^, whereas another study showed a positive association of MVPA with resting BDNF and an inverse association of total time spent sitting with resting BDNF^31^. To our knowledge, only Júdice and colleagues examined the relationship between patterns of SB and resting BDNF in individuals with type 2 diabetes and found that prolonged uninterrupted periods in sedentary time (bouts > 15 min), but not total time spent sitting, was negatively associated with resting BDNF^33^.

However, evidence on associations of combined accelerometer-measured patterns of habitual PA and SB with BDNF in adults with CVD is lacking. Therefore, the aim of this study was to analyze the association between accelerometer-measured habitual PA (LPA, MVPA) and SB (total sitting time and different lengths of sedentary bouts) with resting levels of serum BDNF in individuals aged ≥ 60 years and a history of coronary heart disease (CHD).

## Methods

### Study design and participants

For this non-controlled pre-post study, individuals who have had an inpatient treatment within the last 24 months prior to the start of the study (November 2017) at the Department of Internal Medicine B at the University Medicine Greifswald, were recruited. Eligibility criteria for the study participation are shown in Table 1.

**Table 1:** Inclusion and exclusion criteria for study participation.

Inclusion criteria
• Age of ≥ 60 years • Established CVD defined by a stenosis of ≥ 70% of a least one coronary vessel • Optimal medical treatment according to the European Society of Cardiology guidelines (2016)
Exclusion criteria
• Heart failure, left ventricular ejection fraction (LVEF) < 40% • Implanted cardioverter/defibrillator or pacemaker • Recent cardiovascular event ≤ 2 months prior to study inclusion (acute myocardial infarction, resuscitation, re-vascularization, device implantation, or stroke) • Planned coronary revascularization • Uncontrolled blood pressure (systolic blood pressure of ≥ 200 mmHg) • Body mass index ≥ 35 kg/m^2^ • Baseline cardiopulmonary exercise test results precluding safe exercise training (e.g., ischemia or arrhythmias) • No ability to participate in exercise training (e.g., COPD GOLD III-IV, claudication ≥ 2b, or previous disabling stroke) • Current mental disorder requiring inpatient treatment • Current addictions (excluding tobacco use), florid psychoses, current severe depressive episode (according to ICD-10) • Severe cognitive or physical impairment • No serious co-existing diseases (e.g., cancer) with life expectancy < 1 year • Weekly self-reported PA ≥ 150 min on a moderate intensity level ≤ 6 months prior to study inclusion

CVD = Cardiovascular Disease, COPD GOLD III-IV = Chronic Obstructive Pulmonary Disease stage III according to the Golden Initiative for Chronic Obstructive Lung Disease, ICD-10 = International Statistical Classification of Diseases and Related Health Problems 10th Revision, PA = Physical Activity.

A total of 164 individuals with CHD were contacted by mail and invited to participate in the study that aimed to test the feasibility of a multi-behavioral lifestyle intervention. Among those, 127 individuals declined to participate due to several reasons such as (i) a lack of interest (n = 87) or (ii) time and physical demands of intervention or data assessment (n = 24 too ill, n = 8 barriers to reach the training- and cardiovascular examination center, n = 6 heavy work schedules, n = 2 other barriers). One individual agreed to participate, but did not complete enrolment because of severe illness. Thus, the final sample comprised 36 individuals.

The focus of the lifestyle intervention was PA and nutrition. Views on ageing as a psychological component formed the framework of the intervention^35^. The program of the intervention consisted of group and individual sessions and took part at the cardiovascular training and examination center of the University Medicine Greifswald for a duration of 12 weeks. Participants exercised twice a week (from week 1 to 6) or once a week (from week 6 to 12) in a group setting, respectively (overall: 18 sessions). Views on ageing were discussed with the participants in three group sessions and one individual session. Furthermore, participants received four education group sessions focusing on healthy eating from week 6 to 11. At week 6 and 12, an individual session was carried out in order to receive process information on participants’ motivation, study quality, and potential for optimization of the study. From week 12 to 24, participants had the opportunity to exercise once a week in a group at the cardiovascular examination center on a voluntary basis. Each session lasted 60 to 90 min.

This study was conducted in accordance with the Declaration of Helsinki and current guidelines of good clinical practice. The ethics committee of the University Medicine Greifswald approved the study protocol (number BB138/17). Written informed consent was obtained before inclusion of study participants.

### Procedures

This study is a secondary analysis of baseline data of an intervention study (“ReStart 60 + ”) with a duration of 12 months. All participants were invited to the cardiovascular training- and examination center of the University Medicine Greifswald and underwent the following procedure: (i) standardized measurement of anthropometrical data, (ii) blood collection, (iii) resting electrocardiogram recording, and (iv) self-administered assessments. Detailed information on current medical status and medications was collected. Body weight and height as well as waist and hip circumference were assessed. Waist circumference was measured midway between the lowest rib and the iliac crest using an inelastic tape. Hip circumference was measured about two inches below the iliac crest. Blood samples were drawn in the supine position from participants who gave additional informed consent. The sampling was carried out between 7:30am and 1:00 pm. Study participants provided fasting (> 8 h) or non-fasting blood samples. Immediately after sampling, the serum tubes were cooled down to 4 °C. An hourly transport to the central laboratory (Institute for Clinical Chemistry and Laboratory Medicine, University Medicine Greifswald) was organized. Upon arrival at the laboratory, the samples were immediately stored at −80 °C in the Integrated Research Biobank (LiCONiC, Lichtenstein). Trained and certified study staff performed all measurements and blood sampling based on established standard operating procedures.

The day following the cardiovascular examination program, the accelerometer had to be worn for ten consecutive days. Study participants were instructed to wear the accelerometer on their hip with an elastic band after getting dressed in the morning and to take the device off for night’s sleep and water-based activities. The study was conducted between November 2017 and September 2019.

### Measures

#### Serum brain-derived neurotrophic factor concentration

To determine the resting serum levels of BDNF, human serum samples were diluted 1:20 with the appropriate buffer. Human serum BDNF concentrations were measured in duplicates using enzyme-linked immunosorbent assay (ELISA) kit according to the instructions of the manufacturer (Quantikine Human Free BDNF ELISA kit, R&D systems, Minneapolis, MN). Using this assay the minimum detectable dose of human free BDNF is less than 20 pg/ml. The intra-assay coefficient of variability lies between 3.8% and 6.2% while the inter-assay precision is between 7.6 and 11.3%. BDNF levels were calculated using a standard curve supplied by the ELISA kit.

#### Accelerometer-measured physical activity and sedentary behavior

The accelerometers were initialized at a sampling rate of 30 Hertz and raw data were integrated into 60-s epochs^36^. Data from the vertical axis were used. For statistical analysis, data from the accelerometers were downloaded and processed using ActiLife software (Version 6.13.3; ActiGraph). Time spent in SB, LPA, MVPA, and accelerometer wear time was determined by minutes per day (min/d). Non‐wear time was defined as at least 60 consecutive minutes of zero activity counts, with allowance for ≤ 2 counts per minute (cpm) between 0 and 100. We used cut points according to different intensity threshold criteria. Values < 100 cpm were defined as SB, values between 100 and 1951 cpm as light PA, and values ≥ 1952 cpm as MVPA^37^.

In order to minimize measurement bias of reactivity^38^ and due to missing values, we decided not to include data of the first, second, and last accelerometer wearing day. Therefore, seven consecutive days of accelerometer wearing were included for data analyses. Further, we analyzed data only among those who wore the accelerometer on ≥ 4 days per week for ≥ 10 h per day (n = 30)^36^.

#### Covariates

Additional variables including sex and age (years) were assessed as covariates by self-administered questionnaire. There is evidence that BDNF levels are associated with body weight^39^. Thus, statistical models were adjusted for body mass index (BMI) which was calculated as body weight divided by height squared (kg/m^2^) and used as a continuous variable. Accelerometer-based patterns of SB and PA have shown to be associated with accelerometer wear time^40^ and therefore, wear time was included as a covariate.

### Statistical analysis

The model assumptions for normality and homoscedasticity of residuals were tested using Shapiro–Wilk tests, White tests and graphic methods (histograms, kernel density plots, Q-Q plots, residual-versus-fitted plots). The distributions of SB, LPA, MVPA, and accelerometer wear time approximated normality. Thus, untransformed values were used for analyses and participant characteristics were described in means (M) with standard deviations (SD). Estimated daily averages of time spent in MVPA were calculated as total minutes of MVPA (min/d). Estimated daily averages of time spent in LPA were calculated as total minutes of LPA (min/d). Estimated daily averages of time spent in SB were calculated as (i) total minutes of SB (min/d); (ii) number of 10-to-30 min bouts and of > 30 min bouts of SB per day, and (iii) average length of 10-to-30 min bouts and > 30 min bouts of SB.

Separate multiple linear regression models were applied for each accelerometer-based measure (time in SB, LPA, MVPA, SB bouts: numbers and lengths) with resting levels of serum BDNF as outcome variable. In a first step, all analyses were adjusted for potential confounders (model 1: sex, age, BMI, and accelerometer wear time). In a second step, we additionally adjusted for SB in all regression models that included PA variables (e.g., MVPA). However, due to multicollinearity, we did not adjust these models for other PA variables (e.g., LPA). Likewise, all regression models including SB were additionally adjusted for MVPA (model 2). We used robust standard errors estimations to account for heteroscedasticity. *P*-values < 0.05 were considered statistically significant. All statistical analyses were conducted using STATA version 15.1 (Stata-Corp, 2015).

### Ethics approval and consent to participate

All procedures performed in studies involving human participants were in accordance with the ethical standards of the institutional and/or national research committee and with the 1964 Helsinki Declaration and its later amendments or comparable ethical standards. Further, this study was conducted in accordance with the current guidelines of good clinical practice. The ethics committee of the University Medicine Greifswald approved the study protocol (number BB 138/17). Written informed consent was obtained before inclusion of study participants.

### Consent for publication

Participants cannot be individually identified from data published in this manuscript. Participants were made aware of the intent to publish this data when providing informed consent.

## Results

Table 2 provides descriptive characteristics of the study sample (n = 30). The mean age was 69.5 years and 80% were males. The majority of the participants attended school for more than 10 years (58.6%), lived in a partnership (86.7%), and perceived their health as very good or good (53.8%). In total, 18 individuals wore the accelerometer for seven valid days (60.0%), *n* = 8 for six valid days (26.7%), *n* = 3 for five valid days (10.0%), and *n* = 1 for four valid days (3.3%) with an average accelerometer wear time of over 13 h per day.

**Table 2:** Descriptive characteristics of the study sample (n = 30).

	N	Participants
**Socio-demographics**		
Age (years)	30	69.5 ± 6.6
Sex, men	30	24 (80.0%)
School education, > 10 years	29	17 (58.6%)
Partnership, yes	30	26 (86.7%)
**Health**		
Brain-derived neurotrophic factor (ng/ml)	29	48.7 ± 10.2
Smoking, yes	29	2 (6.9%)
Body mass index (kg/m^2^)* < 25 ≥ 25 and < 30 ≥ 30	30	29.5 ± 5.2 4 (13.3%) 15 (50%) 11 (36.7%)
Self-perceived health Poor or very poor Fair Good or very good	26	2 (7.7%) 10 (38.5%) 14 (53.8%)
Vascular intervention (e.g., stent), yes	30	27 (90%)
Medication CHD treatment (e.g., Antihypertensives) Statins Antidiabetic Antidepressants	30	30 (100%) 28 (93.3%) 6 (20.0%) 2 (6.7%)
**Adherence to wearing protocol**		
Valid days of wearing, 7 days	30	18 (60.0%)
Accelerometer wear time (min/d)	30	788.4 ± 77.5
**Physical activity**		
Light physical activity (min/d)	30	258.5 ± 71.2
Moderate-to-vigorous physical activity (min/d)	30	21.2 ± 15.2
**Sedentary behavior**		
Sedentary time (min/d)	30	508.7 ± 76.5
Bouts analysis of sedentary behavior (10—30 min) Average number per day Average length (min)	30	15.5 ± 3.2 22.2 ± 2.1
Bouts analysis of sedentary behavior (> 30 min) Average number per day Average length (min)	30	3.4 ± 1.2 43.8 ± 2.4

CHD = Coronary Heart Disease. Data are presented as *n (%)* for categorical variables and mean (*M*) ± standard deviation (*SD*) for continuous variables, respectively. Data refer to the participants with valid accelerometer wearing days, only (≥ 4 days/ ≥ 10 h/d) (n = 30). * Body mass index was calculated as body weight divided by height squared (kg/m^2^).

**Figure 1:**
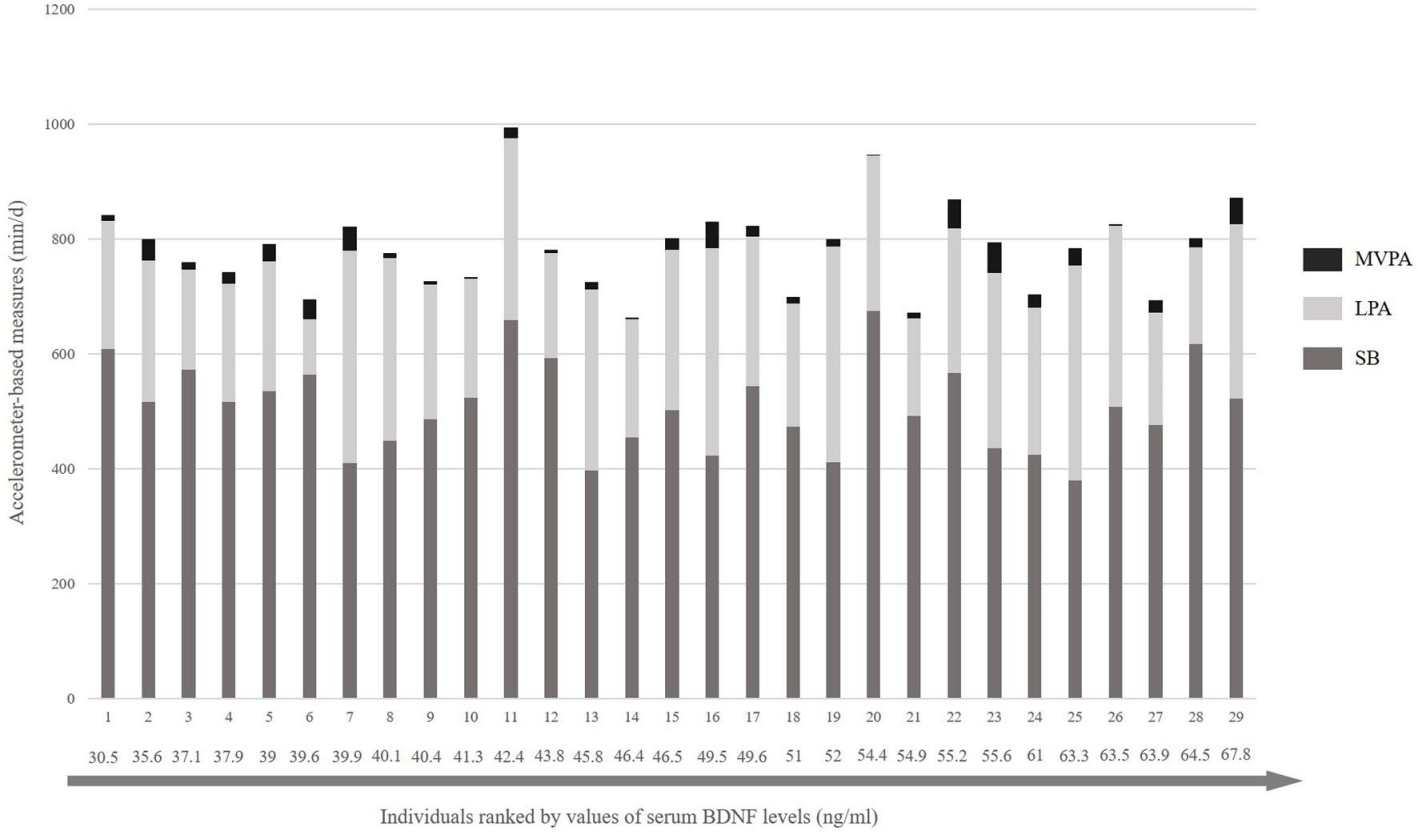
Total time spent in sedentary behavior, light physical activity, and moderate-to-vigorous physical activity among individuals with coronary heart disease (n = 29). Note. SB = Sedentary behavior, LPA = Light physical activity, MVPA = Moderate-to-vigorous physical activity, BDNF = Brain-derived neurotrophic factor. The dark grey segments represent the time spent in SB, the light grey segments represent the time spent in LPA, and the black segments represent the time spent in MVPA. Individuals are ranked by values of BDNF (ng/ml).

Participants spent 508.7 min/d ± 76.5 min/d in SB, which corresponds to 64.5% of accelerometer wear time, 258.5 min/d ± 71.2 min/d in LPA (32.8%), and 21.2 min/d ± 15.3 min/d in MVPA (2.7%). Accelerometer-based measures showed a large inter-individual variability across the 7-day-assessment (Appendix, Fig. S1). Individuals had a daily number of 15.5 ± 3.2 10-to-30-min bouts of SB with an average length of a single bout of 22.2 ± 2.1 min. Further, they had a number of 3.4 ± 1.2 of > 30 min bouts of SB with an average length of 43.8 ± 2.4 min per single bout. The mean of the resting levels of serum BDNF was 48.7 ± 10.2 ng/ml. Figure 1 displays the total time spent in SB, LPA, and MVPA in individuals with CHD ranked by serum BDNF values.

As shown in Table 3, regression analyses in 29 study participants revealed that there were no statistically significant associations between any of the accelerometer-based measures with serum levels of BDNF either in model 1 or model 2 (all *P*-values > 0.05).

**Table 3:** Results of separate linear regression models for minutes spent in light physical activity, moderate-to-vigorous physical activity, and sedentary behavior as well as numbers and average length of 10–30 min and > 30 min sedentary bouts with serum brain-derived neurotrophic factor levels (n = 29).

BDNF	Model 1^a^		Model 2^b^	
	b	(95% CI)	b	(95% CI)
LPA: Total time	0.03	(−0.03; 0.09)	−0.13	(−0.50; 0.23)
MVPA: Total time	0.15	(−0.21; 0.52)	0.13	(−0.23; 0.50)
SB: Total time	−0.04	(−0.09; 0.02)	−0.04	(−0.09; 0.02)
SB: 10—30 min bouts, Number	−1.05	(−2.42; 0.32)	−1.05	(−2.45; 0.34)
SB: 10—30 min bouts, Average length	0.78	(−1.42; 2.98)	0.98	(−1.58; 3.55)
SB: > 30 min bouts, Number	−0.69	(−4.44; 3.06)	−0.34	(−4.61; 3.93)
SB: > 30 min bouts, Average length	0.24	(−1.36; 1.85)	0.23	(−1.43; 1.89)

BDNF = Brain-derived neurotrophic factor, LPA = Light physical activity, MVPA = Moderate-to-vigorous physical activity, SB = Sedentary behavior, b = unstandardized regression coefficient, CI = Confidence Interval. ^a^ Adjusted for sex, age, body mass index, and accelerometer wear time. ^b^ Additionally adjusted for SB in the regression models that included physical activity variables (LPA, MVPA). Regression models including SB were additionally adjusted for MVPA.

## Discussion

In this study, we examined the association of accelerometer-measured habitual PA (LPA, MVPA) and SB (total sitting time as well as numbers and different length of sedentary bouts) with resting levels of serum BDNF among individuals with CHD. Taken together, our results revealed two main findings: First, individuals with CHD spent most of their day in SB and little time in MVPA. In addition, the distribution of time spent in different accelerometer-based measures showed a large inter-individual variability. Second, we could not reveal a statistically significant associations of accelerometer-based measures with resting levels of serum BDNF.

In line with previous research, we found a pattern characterized by low levels of MVPA and high levels of SB among individuals with CHD^14,16^, despite the fact that sample characteristics of these studies such as age (mean age: 63 years;^14^ mean age: 79 years^16^) and proportion of sex (e.g., only women^16^) differed notably from our study sample. Furthermore, our results revealed a large inter-individual variability in daily accelerometer-based measures. It could be speculated that this is one of the reasons why we did not detect an association between accelerometer-based measures and resting levels of serum BDNF. Further research is needed to clarify the effects of different intensities of habitual PA and SB accumulation pattern measures on levels of serum BDNF in individuals with CHD.

Assuming that lifestyle behaviors such as habitual PA and SB can influence cardio-protective factors such as BDNF in individuals with CHD, it might be considered as a strategy to maintain or increase physical and brain health status. As pointed out in a systematic review^9^ and a meta-analysis^7^, studies regarding accelerometer-measured habitual PA and SB and resting BDNF in non-healthy adults (e.g., obesity, CVD, diabetes) are limited and pattern variables of PA and SB (e.g., sedentary bouts) were rarely reported in these studies. Only the study by Júdice and colleagues examined accelerometer-measured habitual PA and patterns of SB and resting BDNF in adults with type 2 diabetes mellitus (mean age: 58.3 years)^33^ which represents also a vulnerable group because these individuals are less active and have lower levels of BDNF^41^. In line with their results, we could not find an association between habitual MVPA and resting BDNF. It should be noted that there is no scientific consensus on how habitual PA or SB and resting levels of serum BDNF might be related and what the biological mechanism might be. Some have argued that resting levels of serum BDNF only increase temporarily after acute bouts of PA, in an intensity- and duration-dependent manner^10^, and that BDNF levels return to pre-PA concentrations after 15 up to 60 min following PA cessation^42,43^. Therefore, it seems to be important to distinguish evidence that reported effects of an acute bout of PA on the one hand and the effects of habitual PA on resting levels of BDNF on the other hand.

In contrast to our results, Júdice and colleagues found that the number of sedentary breaks were positively associated with plasma BDNF concentration, whereas number of longer sedentary bouts per day (> 15 min) were negatively associated with plasma BDNF concentration^33^. Although sedentary breaks and sedentary bouts describe two different pattern parameters and are therefore not interchangeable, both define the accumulation pattern of sitting time and are interdependent^19^. If individuals interrupt their total sitting time often (i.e., having many breaks), the number of short sedentary bouts would increase and the number of longer sedentary bouts would decrease. Our findings may be different from those reported by Júdice and colleagues due to methodological aspects such as sample characteristics (e.g., age, medical condition), accelerometer data processing criteria (e.g., 15-s epochs versus 60-s epochs), operationalization of SB pattern measures (e.g., prolonged sedentary bouts > 15 min versus > 30 min), or BDNF measurement methods (e.g., plasma versus serum BDNF; different ELISA kits). Given that also measurement errors in biological assays may occur due to serum collection or different methodological approaches^44^, the results of different studies on serum and plasma BDNF levels might not be comparable^45^.

Various strengths and limitations should be noted regarding the study design, study sample characteristics, and methodological issues. To our knowledge, the present study is the first one that examined associations of accelerometer-measured habitual PA and SB with levels of serum BDNF in individuals with CHD. Further, it promotes the understanding of patterns of activity, inactivity, and SB accumulation in individuals with CHD by combining information on duration of different PA intensities and SB measures (i.e., sedentary bouts). However, several limitations have to be discussed. First, individuals were assessed within a pre-post study aiming to test the implementation of a multi-modal intervention program in individuals with CHD. The sample size was small. This might have limited the possibility to detect associations between accelerometer-based measures and serum BDNF (i.e., decreased statistical power). Further, the proportion of individuals who declined to participate (78%) was high, which may have led to a sample selection bias. Therefore, generalizability of our results may be compromised. Secondly, there are methodological limitations regarding the measurement of BDNF^44,45^ and of accelerometer-based PA and SB^46^. We used hip-worn accelerometers that may not accurately measure all types of PA (e.g., bicycling) or miss activities (e.g., swimming). Moreover, accelerometers cannot differentiate well enough between sitting and standing because movement is determined by acceleration rather than body posture.^47^ Given that there are no validation or calibration studies in individuals with CVD^48^, we used the recommendations for data collection and analysis that were developed for general populations^36^. Thirdly, there may be confounding variables that influence the association of habitual PA and SB and BDNF, such as medications (e.g., antidepressants) or other lifestyle behaviors (e.g., smoking, diet, alcohol) that were not considered in the current analysis. Fourthly, the proportion of male participants was high (80%). Although study participants with CHD in PA intervention studies appear to be more frequently male (69%)^49^, the low number of females did not allow to stratify our results by sex. Recent evidence demonstrated that BDNF is a sexually dimorphic neurotrophin^50^ and Schmalhofer and colleagues have shown that there were significant sex-specific associations between serum BDNF and cardiorespiratory fitness in women but not in men^51^. Therefore, the findings of our study should be interpreted cautiously and verified in a larger sample of individuals with CHD using stratification by sex. Finally, due to the cross-sectional character of the study results, it is not allowed to drawn inferences about any possible causal associations between the predictor and outcome variables.

## Conclusion

To conclude, the cross-sectional findings of our study suggest that pattern variables of accelerometer-measured PA and SB seems not to be related with serum BDNF in individuals with CHD. In addition, our data show a substantial inter-individual variability of habitual PA and SB. Given that this study is one of the first to examine the association between different habitual accelerometer-based measures and serum BDNF, further research is needed to confirm our results.

## Supplementary Information

Supplementary Information.

## Data Availability

The datasets generated and/or analyzed during the current study are not publicly available due to restrictions associated with anonymity of participants but are available from the corresponding author on reasonable request. The data is shared with researchers who submit a methodologically sound proposal to achieve the aims of the approved proposal., Requests in this regard should be directed to the corresponding author to gain access. Requestors must sign a data access agreement ensuring data usage in compliance with the statement given in the informed consent procedure and with the German data protection law, that the data will not be transferred to others, and that the data will be deleted after the intended analysis has been completed.
