# Supplementary material for: Preliminary results of the cross-sectional associations of sedentary behavior and physical activity with serum brain-derived neurotrophic factor in adults with coronary heart disease
Source: Sci Rep. 2022 Nov 16;12:19685. doi: 10.1038/s41598-022-23706-8 (PMC9669050; doi:10.1038/s41598-022-23706-8)
Supplement: Supplementary file 1 — Supplementary Information. [file 41598_2022_23706_MOESM1_ESM.docx]

*Appendix*

**
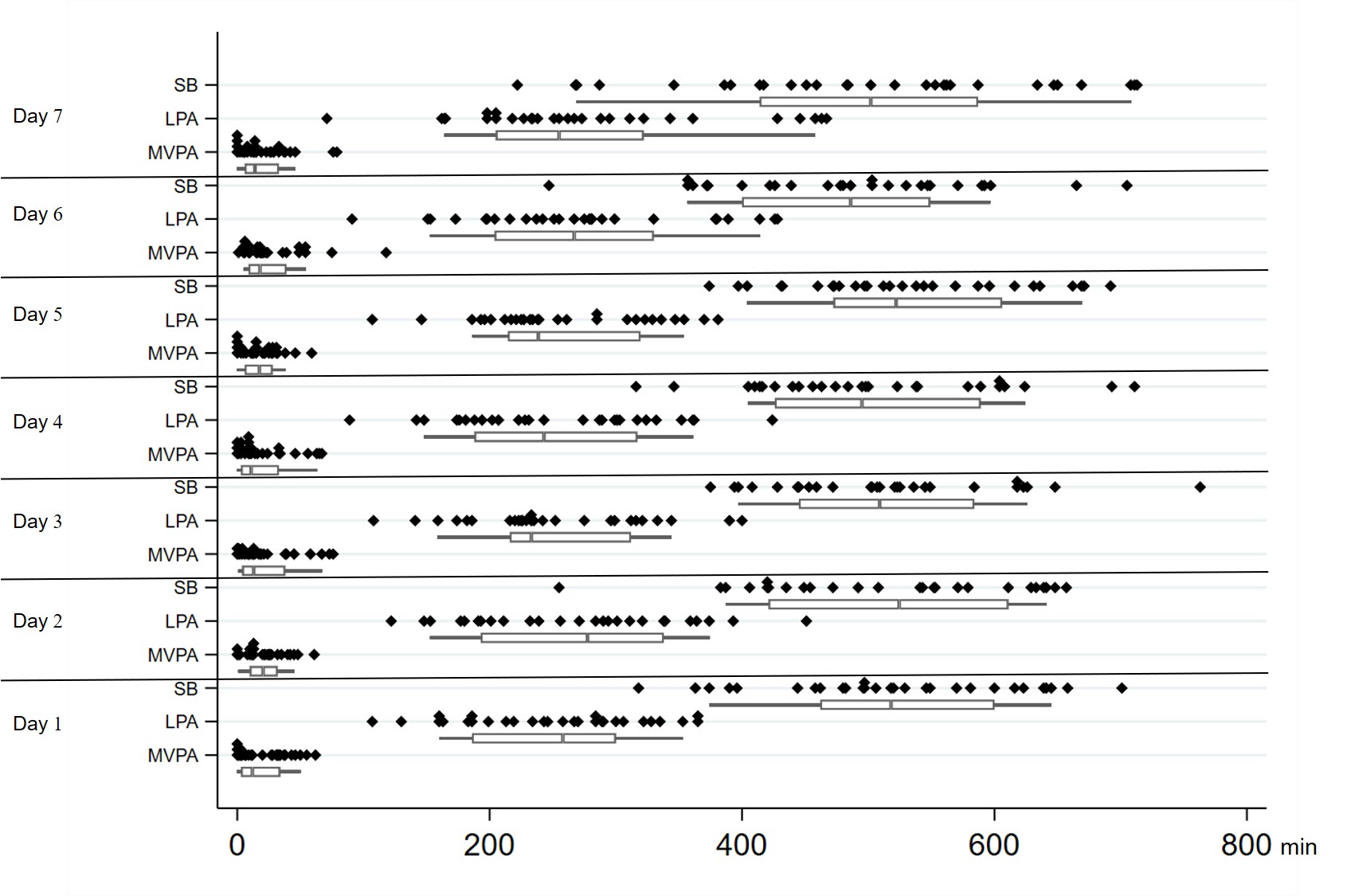
**

**Fig. S1** Distribution of time spent in accelerometer-based measures by plotting individual data over each day of the wearing period (stripplot with boxplots, n = 30)

Note. SB = Sedentary behavior, LPA = Light physical activity, MVPA = Moderate-to-vigorous physical activity.
